# Supplementary material for: Optimizing Ghanaian Postgraduate students’ job performance: The impact of polychronicity, work-school facilitation, and organisational support
Source: PLoS One. 2025 Sep 18;20(9):e0329070. doi: 10.1371/journal.pone.0329070 (PMC12445522; doi:10.1371/journal.pone.0329070)
Supplement: S1 Appendix — (DOCX) [file pone.0329070.s002.docx]

**Data for article**

APPENDIX A

ITEMS

Please provide your candid responses using a scale of 1 to 5. With 1 = Least Agreement (LD) 2= Disagree (D), 3 = Neutral (N), 4 = Agree (A), 5 = Strongly Agree (SA) *OR* 1 (least agreement) to 5 (highest agreement), rate your level of disagreement *or* agreement with itemized questions under each variable by ticking (√) in the most appropriate column.

| **N°** | **Item** | **1** | **2** | **3** | **4** | **5** |
| --- | --- | --- | --- | --- | --- | --- |
| **POLYCHRONICITY** | | | | | | |
| 1 | I like to do several things at once |  |  |  |  |  |
| 2 | I can work on more than one task efficiently |  |  |  |  |  |
| 3 | I feel more satisfied when I multitask |  |  |  |  |  |
| 4 | I can easily switch from one task to another |  |  |  |  |  |
| 5 | I can coordinate multiple tasks without losing track of them |  |  |  |  |  |
| **JOB PERFORMANCE** | | | | | | |
| 1 | I have the skills and knowledge required to perform my tasks effectively |  |  |  |  |  |
| 2 | I am satisfied with the quality of my work output |  |  |  |  |  |
| 3 | I have clear and realistic goals and expectations for my work |  |  |  |  |  |
| 4 | I receive constructive feedback and guidance from my supervisor and colleagues |  |  |  |  |  |
| 5 | I contribute positively to the team and the organisation's objectives |  |  |  |  |  |
| **WORK SCHOOL FACILITATION** | | | | | | |
| 1 | I feel supported by my work colleagues in balancing my work and school commitments |  |  |  |  |  |
| 2 | I have access to adequate resources and guidance from my work to help me with my school assignments |  |  |  |  |  |
| 3 | I have a clear career path and advancement plan that aligns with my school aspirations and qualifications |  |  |  |  |  |
| 4 | I can balance my work and school responsibilities without compromising my performance in either domain |  |  |  |  |  |
| 5 | I feel motivated and inspired by my work environment to continue my education and lifelong learning |  |  |  |  |  |
| **ORGANISATIONAL SUPPORT** | | | | | | |
| 1 | My organisation provides me with adequate resources and opportunities to perform well |  |  |  |  |  |
| 2 | My organisation cares about my well-being and satisfaction |  |  |  |  |  |
| 3 | My organisation recognises and rewards my achievements |  |  |  |  |  |
| 4 | My organisation listens to and acts upon my suggestions and feedback |  |  |  |  |  |
| 5 | My organisation treats me fairly and respectfully |  |  |  |  |  |
| 6 | The organisation takes pride in my accomplishments at work. |  |  |  |  |  |

Top of Form
