# Supplementary material for: Optimizing Ghanaian Postgraduate students’ job performance: The impact of polychronicity, work-school facilitation, and organisational support
Source: PLoS One. 2025 Sep 18;20(9):e0329070. doi: 10.1371/journal.pone.0329070 (PMC12445522; doi:10.1371/journal.pone.0329070)
Supplement: Attachment — Submitted filename: Review PONE-D-24-21809.pdf [file pone.0329070.s004.pdf]

**REVIEW MANUSCRIPT PONE-D-24-21809**

# OPTIMIZING GHANAIAN POSTGRADUATE STUDENTS' JOB PERFORMANCE: THE IMPACT OF POLYCHRONICITY, WORK-SCHOOL FACILITATION, AND ORGANISATIONAL SUPPORT

**Dear Authors,**
I have the opportunity to review this interesting article. Apart from its many advantages, several things need to be enriched and added to make it more perfect as a scientific work, among others:

## Introduction
Add research gaps between variables.

## Literature Review
- Add sufficient narrative or explanation of the conceptual framework.
- According to the conceptual framework, add a hypothesis about the relationship between Work School Facilitation and job performance.

## Methodology
Add references and test for common method bias (CMB) adequately. As a guidances/references, see the link: https://doi.org/10.3390/educsci14040384; https://doi.org/10.3390/jintelligence10030044.

## Results
- Add a Results section with the order of presentation: Description and Correlation Analysis (Mean, Standard Deviation, and Correlation Coefficient of Variables); Measurement Model; Structural model; Fit Model; and Slope Analysis.
- Hypothesis test results should be interpreted adequately, including the addition of new hypotheses (Work School Facilitation with Job Performance).

## Discussion of Hypotheses
- This section should be changed to just "Discussion."
- Add an analysis of the relationship between Work School Facilitation and job performance.
- The discussion for each hypothesis should be sharpened and analyzed more critically, compared with previous research and research gaps.

## Suggestions for future studies
In this section, change it to "Limitation and Future Research." Add limitations to this study and relate them to Future Research.

## Conclusion
Add a conclusion section containing the main research findings. Show originality as a novelty. Complete the theoretical contribution and novelty of the findings.
