# Supplementary material for: Optimizing Ghanaian Postgraduate students’ job performance: The impact of polychronicity, work-school facilitation, and organisational support
Source: PLoS One. 2025 Sep 18;20(9):e0329070. doi: 10.1371/journal.pone.0329070 (PMC12445522; doi:10.1371/journal.pone.0329070)
Supplement: Attachment — Submitted filename: Review PONE-D-24-21809-RI.pdf [file pone.0329070.s006.pdf]

REVIEW MANUSCRIPT PONE-D-24-21809-R1

OPTIMIZING GHANAIAN POSTGRADUATE STUDENTS' JOB PERFORMANCE: THE IMPACT OF POLYCHRONICITY, WORK-SCHOOL FACILITATION, AND ORGANISATIONAL SUPPORT

Dear Authors,
I really appreciate your hard work in fulfilling my comments. Many thanks for it. However, several parts of the article still need improvement. Some of them which are quite crucial are:

The description of CMB is in the Methodology and Results Section—merge them into one. The citation source regarding CMB that you added does not explain CMB at all, so it would be better if you use the article references with the following links: https://doi.org/10.3390/educsci14040384; https://doi.org/10.3390/jintelligence10030044. In addition, it would also be better if you added a description of what CMB is, its urgency, and how to overcome it. Both articles can be used as references.

In Table 7, sort H1 – H5. Hypothesis test results should be interpreted adequately. What do these results mean? All path coefficient values are positive; what does that mean? Also, explain which result is the largest and what its consequences are. Then, it also shows the smallest result along with its consequences.
