# Supplementary material for: Optimizing Ghanaian Postgraduate students’ job performance: The impact of polychronicity, work-school facilitation, and organisational support
Source: PLoS One. 2025 Sep 18;20(9):e0329070. doi: 10.1371/journal.pone.0329070 (PMC12445522; doi:10.1371/journal.pone.0329070)
Supplement: Attachment — Submitted filename: OPTIMISING POSTGRADUATE STUDENTS JOB PERFORMANCE THE IMPACT.docx [file pone.0329070.s003.docx]

In fact, this study" OPTIMISING POSTGRADUATE STUDENTS' JOB PERFORMANCE: THE IMPACT OF POLYCHRONICITY, WORK SCHOOL FACILITATION AND ORGANISATIONAL

SUPPORT" came with an attractive and important topic, and the important variables were linked in a logical and purposeful way. This link led to reaching a logical model for the study. Also, the study problem and research gaps were clearly and appropriately shown. Also, the elements of the study were completed in a good and appropriate way, but I have some notes and suggestions.

1- The introduction is long. I suggest abbreviating and emphasizing the elements of a sound introduction, which is to summarize the previous efforts of previous researchers, then what is the gap, then what have we done in this aspect, then what is the value and addition that this effort can provide.
2- Suggest to the author(s) to enhance the literature supporting the hypotheses of why the relationship occurred and what outcome occurred as a result of this relationship.
3- After presenting the literature supporting the hypotheses and presenting the model, which is a clear model, I suggest that the author write a paragraph that integrates all the relationships, a paragraph that shows how the complete structure of the model. I think that this will enhance that integrated model.
4- In methodology, the sample design and the method of obtaining and collecting data from it were clear and precise, but I suggest here explaining how it can contribute to generalizing the results to other student communities.
5- In **the Ethics Statement** section, the author states that the sample was targeted at postgraduate students over the age of 18. I think postgraduate students typically start from 21 or 22, so I suggest revising this note.
6- In the **Measurement** section, it would be better to mention the number of items.

used for each variable, with specific references.

7- I did not see at the end of the study a reference to the study’s limitations, which is an important matter in the research that clarifies to the reader the aspects that the study did not cover without affecting the value of the results. Also, there is a reference to the possibility or impossibility of generalizing the results.
8- Finally, there is a study on the same topic published in 2022. I suggest that the researcher refer to it and benefit from it for the purposes of this research. It is:

Twaissi, Naseem., Alhawtmeh, Omar. & Al-Laymoun .,O’la (2022) Polychronicity, job performance, and work engagement: The mediating role of supervisor’s organizational embodiment and moderation of psychological ownership, Cogent Business & Management, 9:1, DOI: [10.1080/23311975.2022.2143012](https://doi.org/10.1080/23311975.2022.2143012)

I believe this study is suitable for publication after making the minor modifications written in this report.
