# Supplementary material for: Optimizing Ghanaian Postgraduate students’ job performance: The impact of polychronicity, work-school facilitation, and organisational support
Source: PLoS One. 2025 Sep 18;20(9):e0329070. doi: 10.1371/journal.pone.0329070 (PMC12445522; doi:10.1371/journal.pone.0329070)
Supplement: Attachment — Submitted filename: Response to Reviewers comments.docx [file pone.0329070.s005.docx]

Response to Reviewers comments;

Thank you for your comments regarding our manuscript. In attending to the comments, we used a red color to show places where correction was made in the manuscript.

| Reviewer 1 comments | Response | Page |
| --- | --- | --- |
| The introduction is long. I suggest abbreviating and emphasizing the elements of a sound introduction, which is to summarize the previous efforts of previous researchers, then what is the gap, then what have we done in this aspect, then what is the value and addition that this effort can provide. | The introduction has been improved and reduced to two pages and instead of four and has captured all comments addressed here. | 2-4 |
| After presenting the literature supporting the hypotheses and presenting the model, which is a clear model, I suggest that the author write a paragraph that integrates all the relationships, a paragraph that shows how the complete structure of the model. I think that this will enhance that integrated model | A paragraph has been added after the conceptual framework to integrate all the hypotheses | 9 |
| The author states that the sample was targeted at postgraduate students over the age of 18. I think postgraduate students typically start from 21 or 22, so I suggest revising this note | This has been rectified and the age starts from 21 | 11 |
| In the **Measurement** section, it would be better to mention the number of item used for each variable, with specific references | This has been added under the first paragraph in the measurement section “In this view, 5-items for polychronicity were adapted from the empirical work of (Bluedorn et al., 1999). Again, 5-items for work-school facilitation and -items for organisational support were adapted from (Frone et al., 1992 and Rhoades & Eisenberger, 2002) respectively. And finally, 5-items for job performance were adapted from (Woods, 2008). All items were measured on a 5-item Likert scale ranging from least agreement to high agreement”. | 11-12 |
| I did not see at the end of the study a reference to the study’s limitations, which is an important matter in the research that clarifies to the reader the aspects that the study did not cover without affecting the value of the results. Also, there is a reference to the possibility or impossibility of generalizing the results | Limitations of the study has been added and we have also added some suggestions for future studies | 24 |
|  |  |  |
| **Reviewer 2** |  |  |
| Add research gaps between variables. | The research gaps are capture in between the paragraphs that links explains the variables for instance, in the last but one paragraph in page 2, second paragraph in page 3 and the last paragraph in page 4. These paragraphs show the main gaps that the study is premised on. | 2-4 |
| Add sufficient narrative or explanation of the conceptual framework. | A narration has been provided beneath the framework to explain the relationships among the variables and hypotheses | 9 |
| According to the conceptual framework, add a hypothesis about the relationship between  Work School Facilitation and job performance. | Hypothesis H4 has been created to cater for this | 8 |
| Add references and test for common method bias (CMB) adequately. | Harman’s Single Factor Test has been conducted to cater for CMB and the results has been reported on the last paragraph and last sentence under “data analysis section” | 12 and 13 |
| Add a Results section with the order of presentation: Description and Correlation Analysis  (Mean, Standard Deviation, and Correlation Coefficient of Variables); Measurement Model;  Structural model; Fit Model; and Slope Analysis. | These arrangements have been duly done | 13-20 |
| Hypothesis test results should be interpreted adequately, including the addition of new  hypotheses (Work School Facilitation with Job Performance). | This has been done. Again, a new hypothesis (H4) has been added in the fourth paragraph in the discussion section to cater for the effect of work school facilitation on job performance | 21-22 |
| In this section, change it to "Limitation and Future Research." Add limitations to this study and  relate them to Future Research. | A section on limitation and future research has been added | 24 |
| Add a conclusion section containing the main research findings. Show originality as a novelty.  Complete the theoretical contribution and novelty of the findings. | This has been added | 22-23 |
|  |  |  |
