# Supplementary material for: Optimizing Ghanaian Postgraduate students’ job performance: The impact of polychronicity, work-school facilitation, and organisational support
Source: PLoS One. 2025 Sep 18;20(9):e0329070. doi: 10.1371/journal.pone.0329070 (PMC12445522; doi:10.1371/journal.pone.0329070)
Supplement: Attachment — Submitted filename: Final Review of the Paper.docx [file pone.0329070.s007.docx]

**Subject:** Final Review of the Paper

Dear Editor,

I appreciate the recent amendments, which have positively enhanced the paper. Based on these improvements, I find the paper suitable for publication.

However, I would like to bring your attention to a minor issue in the reference list. Reference 13 is formatted inconsistently compared to the other references. Could you please ensure that it follows the same format as the rest?

Best regards,
