# Supplementary material for: Optimizing Ghanaian Postgraduate students’ job performance: The impact of polychronicity, work-school facilitation, and organisational support
Source: PLoS One. 2025 Sep 18;20(9):e0329070. doi: 10.1371/journal.pone.0329070 (PMC12445522; doi:10.1371/journal.pone.0329070)
Supplement: Attachment — Submitted filename: Response to Reviewers comments 1.1.docx [file pone.0329070.s008.docx]

Response to Reviewers comments;

Thank you for your comments regarding our manuscript. In attending to the comments, we used a red color to show places where correction was made in the manuscript.

| Editor’s comments | Response | Page |
| --- | --- | --- |
| The Harman’s single-factor test reveals common method bias with a variance of 44.13%, which is below the recommended threshold, potentially distorting relationships between variables and affecting internal validity. What measures did you use to reduce such bias. | Thank you for your comment, however, it has been established that the acceptable threshold should be less than 50%, and from this paper, the acceptable threshold was met. This shows that there were no issues of biases. *See; Tehseen et al. (2017) and Kock, (2020) as cited by Damanik and Widodo, (2024)* | Las paragraph on page 15 |
| Retaining weak indicators with factor loadings below 0.4 compromises construct validity and reliability. Please explain why specific indicators (e.g., OS6) were kept despite lower factor loadings. | According to Hair et al. (2014, 2016), Jo, & Jeon, (2021) and Gefen et al. (2000) indicator loading of 0.4 and above are acceptable provided it does not affect the AVEs and structural path. And this justification were given in the work before retaining (OS6). | Last but one paragraph on 13 |
| The use of convenience sampling introduces bias, which may limit the generalizability of the findings and affecting external validity. Please justify the use of convenience sampling and highlight practical constraints. | While convenience sampling introduces some potential bias, it is often impractical to use randomized sampling in postgraduate research due to accessibility challenges and time constraints [73]. As a result, this study utilized convenience sampling to accommodate students' varied schedules, ensuring participation from those who were available and willing. To address concerns about bias, participants were selected from diverse academic backgrounds. [73] argues that convenience sampling is acceptable in PLS-SEM because it emphasizes predictive accuracy. Additionally, bootstrapping was applied to ensure robust parameter estimation, further mitigating potential biases and enhancing the reliability of the findings. | Last but one paragraph 11 |
| Please justify the appropriateness of the sample size by explaining why the use of Krejcie and Morgan's formula is suitable for this particular study. | Krejcie and Morgan’s formula is suitable for this study as it ensures a statistically reliable sample size for a finite population, maintaining a 95% confidence level with a 5% margin of error. It enhances generalizability by capturing variations within the post-graduate students while balancing practicality and ethical considerations. The formula is widely accepted in social science research as it helps prevent unnecessary over- or under-sampling [72]. Thus, the chosen sample size of 341 is appropriate for drawing valid conclusions. | First paragraph 10 |
|  |  |  |
| **Reviewer 1** |  |  |
| I would like to bring your attention to a minor issue in the reference list.  Reference 13 is formatted inconsistently compared to the other references. Could you please ensure that it follows the same format as the rest? | Twaissi, N. M., Alhawtmeh, O. M., & O’la Hmoud Al-Laymoun. (2022). Polychronicity, job performance, and work engagement: The mediating role of supervisor’s organizational embodiment and moderation of psychological ownership. *Cogent Business & Management*, *9*(1), 2143012. | 25 |
|  |  |  |
| **Reviewer 2** |  |  |
| The description of CMB is in the Methodology and Results Section—merge them into one.  The citation source regarding CMB that you added does not explain CMB at all, so it would be better if you use the article references with the following links:https://doi.org/10.3390/educsci14040384; https://doi.org/10.3390/jintelligence10030044. In  addition, it would also be better if you added a description of what CMB is, its urgency, and  how to overcome it. Both articles can be used as references. | These references have been used to explain CMB. Widodo, W., Gustari, I., & Chandrawaty, C. (2022). Adversity quotient promotes teachers’ professional competence more strongly than emotional intelligence: Evidence from Indonesia. Journal of Intelligence, 10(3), 44.  Damanik, J., & Widodo, W. (2024). Unlocking Teacher Professional Performance: Exploring Teaching Creativity in Transmitting Digital Literacy, Grit, and Instructional Quality. *Education Sciences*, *14*(4), 384. Fuller, Christie M., Marcia J. Simmering, Guclu Atinc, Yasemin Atinc, and Barry J. Babin. "Common methods variance detection in business research." *Journal of business research* 69, no. 8 (2016): 3192-3198.  Tehseen, S., Ramayah, T., & Sajilan, S. (2017). Testing and controlling for common method variance: A review of available methods. Journal of management sciences, 4(2), 142-168.  Kock, N. (2020). Harman’s single factor test in PLS-SEM: Checking for common method bias. Data Analysis Perspectives Journal, 2(2), 1-6.  Spector, P. E., Rosen, C. C., Richardson, H. A., Williams, L. J., & Johnson, R. E. (2019). A new perspective on method variance: A measure-centric approach. Journal of Management, 45(3), 855-880.\ | Last paragraph on page 14 |
| In Table 7, sort H1 – H5. Hypothesis test results should be interpreted adequately. What do  these results mean? All path coefficient values are positive; what does that mean? Also, explain  which result is the largest and what its consequences are. Then, it also shows the smallest result along with its consequences. | This section has been improved to suit your comment | 18-20 |
|  |  |  |
