# Supplementary material for: Optimizing Ghanaian Postgraduate students’ job performance: The impact of polychronicity, work-school facilitation, and organisational support
Source: PLoS One. 2025 Sep 18;20(9):e0329070. doi: 10.1371/journal.pone.0329070 (PMC12445522; doi:10.1371/journal.pone.0329070)
Supplement: Attachment — Submitted filename: Response to the journal comments.docx [file pone.0329070.s009.docx]

Response to the journal comments. Actions taken are written in red ink in the manuscript.

Please review your reference list to ensure that it is complete and correct.

All references have been provided, and all omissions have been rectified.

For instance, all these references which were omitted have been provided in the revised manuscript.

1. Twaissi, N. M., Alhawtmeh, O. M., & O’la Hmoud Al-Laymoun. (2022). Polychronicity, job performance, and work engagement: The mediating role of supervisor’s organizational embodiment and moderation of psychological ownership. *Cogent Business & Management*, *9*(1), 2143012.
2. Widodo, W., Gustari, I., & Chandrawaty, C. (2022). Adversity quotient promotes teachers’ professional competence more strongly than emotional intelligence: Evidence from Indonesia. Journal of Intelligence, 10(3), 44.
3. Damanik, J., & Widodo, W. (2024). Unlocking Teacher Professional Performance: Exploring Teaching Creativity in Transmitting Digital Literacy, Grit, and Instructional Quality. *Education Sciences*, *14*(4), 384.
4. Fuller, Christie M., Marcia J. Simmering, Guclu Atinc, Yasemin Atinc, and Barry J. Babin. "Common methods variance detection in business research." *Journal of business research* 69, no. 8 (2016): 3192-3198.
5. Tehseen, S., Ramayah, T., & Sajilan, S. (2017). Testing and controlling for common method variance: A review of available methods. Journal of management sciences, 4(2), 142-168.
6. Kock, N. (2020). Harman’s single factor test in PLS-SEM: Checking for common method bias. Data Analysis Perspectives Journal, 2(2), 1-6.
7. Spector, P. E., Rosen, C. C., Richardson, H. A., Williams, L. J., & Johnson, R. E. (2019). A new perspective on method variance: A measure-centric approach. Journal of Management, 45(3), 855-880.\
8. Singh, A. S., & Masuku, M. B. (2014). Sampling techniques & determination of sample size in applied statistics research: An overview. International Journal of Economics, Commerce and Management, 2(11), 1-22.
9. Hossan, D., Dato’Mansor, Z., & Jaharuddin, N. S. (2023). Research population and sampling in quantitative study. International Journal of Business and Technopreneurship (IJBT), 13(3), 209-222.
