# Supplementary material for: Optimizing Ghanaian Postgraduate students’ job performance: The impact of polychronicity, work-school facilitation, and organisational support
Source: PLoS One. 2025 Sep 18;20(9):e0329070. doi: 10.1371/journal.pone.0329070 (PMC12445522; doi:10.1371/journal.pone.0329070)
Supplement: Attachment — Submitted filename: Response to the journal comments (1)-3.docx [file pone.0329070.s010.docx]

Response to the journal comments. Actions taken are written in red ink in the manuscript.

Please review your reference list to ensure that it is complete and correct.

All references have been provided, and all omissions have been rectified.

For instance, all these references that were omitted have been provided in the revised manuscript.

| *Comments* | *Response* | *Page* |
| --- | --- | --- |
| **Reviewer 4** |  |  |
| The abstract and keywords do not provide good information about the article. It is not clear what the main objective of the research is. | The abstract and keywords section has been rewritten to reflect the study’s purpose and objectives | See page 1 |
| The article has problems with clarity and coherence in its language, which need to be reviewed. The Introduction does not make it clear what the objective of the research is, and what the research question (or questions) to be answered in the study are. | The objectives of the study have been provided in the introduction | See the last paragraph of the introduction section on page 4 |
| **Reviewer 5** |  |  |
| For reliability, McDonald Omega can be given in addition to Cronbach's Alpha. | The McDonald Omega reliability test has been conducted with the help of the indicator loadings for each construct. | See page 16 and Table 3 |
| Tolerance and CI can be given for the multicollinearity problem | Tolerance = 1−R^2^ (for each predictor in the regression). Since the researchers used PLS-SEM and not all R^2^ values for predictors are reported here, we typically used VIF (Variance Inflation Factor). However, the CI values are reported in Table 7. | See page 19 and Table 7 |
| It would be nice if a few more values such as RMSEA, CFI are given as model fit indexes | Good suggestion, however, the researcher used PLS-SEM and not CB-SEM. Thus, the indexes provided are given in PLS-SEM | See page 21 |
| Calculating the effect size of the study would be good for understanding the results reached in the findings | This has been provided | See page 19 and Table 7 |
| Limitations related to the sampling method should be mentioned. | This has been provided | Page 10 in the method section |
| **Reviewer 6** |  |  |
| final light proofread to address minor grammatical points or awkward phrasing would further enhance its readability before publication. | This has been done |  |
| **Editor** |  |  |
| The research gap and significance of the study are not clearly articulated, making it difficult to understand how this work advances existing knowledge. The discussion section also needs substantial revision to present a balanced and critical debate, integrating the study's findings with both supporting and contradictory evidence from the current literature. At present, the discussion lacks depth, critical reflection, and fails to provide meaningful insights into the implications of the results. | The gaps that the study intends to fill are summarized in the last paragraph of the introduction  Again, the entire discussion section has been improved | See the last paragraph of the introduction section on page 4  See the discussion section on pages 22-24 |
|  |  |  |
